# Yeast Fermentation and the Make of Biotechnological Products

**DOI:** 10.3390/microorganisms11061463

**Published:** 2023-05-31

**Authors:** Sergi Maicas

**Affiliations:** Laboratory of Microbial Biotechnology, Department of Microbiology and Ecology, Universitat de València, 46100 Burjassot, País Valencià, Spain; sergi.maicas@valencia.edu; Tel.:+34-96-354-3214

## 1. Introduction

Fermentation is a natural process that has been used for thousands of years by humans to produce a variety of foods and beverages. The process involves the use of microorganisms, especially yeasts and bacteria, to convert sugars and other carbohydrates into organic acids, alcohols, and gases [1,2]. This process has been shown to have numerous health benefits due to the production of bioactive compounds and the improvement of the nutritional quality of fermented products [3]. It can occur through two primary methods, natural or spontaneous fermentation and controlled fermentation. In natural or spontaneous fermentation, the microorganisms responsible for fermentation are present in the environment, and fermentation occurs without any intervention. This method is commonly used in the production of sourdough bread, sauerkraut, and other fermented foods [4]. In contrast, controlled fermentation involves the use of a specific yeast strain and controlling the temperature and other conditions to ensure the desired outcome [5]. This method is commonly used in the production of alcoholic beverages, such as beer and wine, as well as in the production of various fermented foods, such as cheese and yogurt [6]. The use of controlled fermentation has been shown to improve the consistency and quality of the final products, as well as to reduce the risk of contamination by harmful microorganisms [7].

## 2. A Bit of History

Fermented beverages have been an integral part of human culture and history for millennia. Their consumption is particularly common in different cultures worldwide. The ability to create these products has been passed down through generations, using a combination of trial and error and meticulous repetition of ancient practices. In Asia, the consumption of mead, or honey wine, was common around 1500 B.C. This beverage was made by fermenting honey and water with yeast, and it was consumed by the Celts, Saxons, Vikings, Greeks, and other ancient cultures [8,9]. The history of beer is also significant, with the earliest records of beer production dating back to the ancient civilizations of Egypt and Mesopotamia (modern-day Iraq). These civilizations used malted barley to produce beer, which was made by boiling the malted barley in water and then allowing it to ferment with yeast. Beer was also popular in China, where the earliest recorded production of beer dates back to the Shang Dynasty (1600–1046 B.C.) [10,11,12]. The production of wine is a long-standing tradition that dates back thousands of years. Archaeological evidence suggests that wine was produced as far back as 6000 B.C. in the region that is now Georgia, where grapevines were first cultivated. Since then, wine has been an integral part of many different cultures and civilizations, including those of Egypt, Greece, and the Roman Empire. The production of wine involves the fermentation of grape juice, which is rich in natural sugars and nutrients. There are also wines produced from other fruits, such as apples, pears, and berries [13,14,15]. South American empires produced chicha from cereals or fruit, which was made by fermenting corn. Chicha is still popular in South America today, with variations made from different types of fruits and grains. In North America, indigenous peoples made fermented beverages from agave, a type of cactus. These beverages were often used in religious and cultural ceremonies [16,17,18].

## 3. The Process

The production of fermented beverages involves several steps, including the selection of raw materials, the preparation of the ingredients, and the fermentation process. The raw materials used in the production of fermented beverages vary depending on the type of beverage being produced. For example, beer is typically made from malted barley, while wine is produced from grapes [19,20]. The preparation of the ingredients is a crucial step in the production of fermented beverages. For beer, the malted barley is mashed, boiled, and then cooled before the yeast is added. Wine is made by crushing the grapes and allowing the juice to ferment with the yeast. The preparation of the ingredients is critical in ensuring the quality of the final product [21,22]. The fermentation process is the most critical step in the production of fermented beverages. During fermentation, the yeast converts the sugars in the raw materials into alcohol and carbon dioxide. The temperature, pH level, and other factors play a significant role in the fermentation process. For example, beer fermentation occurs at temperatures between 10 and 25 °C, while wine fermentation occurs at slightly higher temperatures between 18 and 30 °C [23,24]. The length of the fermentation process varies depending on the type of beverage being produced. For example, beer fermentation typically lasts between one and two weeks, while wine fermentation can last several months. The longer the fermentation process, the more complex the flavor profile of the final product [25,26].

In this Special Issue of the journal *Microorganisms*, Yeast Fermentation 2.0, we aim to provide a platform for articles that focus on the involvement of yeasts in fermentative processes. More specifically, we are interested in studies that investigate yeasts’ roles in the production of fermented products that are of significant value to consumers in both the clinical and food sectors. This Special Issue is a continuation of the previous issue published in 2020, which explored similar topics related to yeast involvement in fermentative processes [27]. By highlighting the latest research findings in this field, we hope to contribute to a better understanding of the crucial role that yeasts play in the production of fermented products that are important for human health and well-being.
